# Application of Microsatellite Loci for Molecular Identification of Elite Genotypes, Analysis of Clonality, and Genetic Diversity in Aspen* Populus tremula* L. (Salicaceae)

**DOI:** 10.1155/2015/261518

**Published:** 2015-12-28

**Authors:** Dmitry V. Politov, Maryana M. Belokon, Yuri S. Belokon, Tatyana A. Polyakova, Anna V. Shatokhina, Elena A. Mudrik, Anna B. Azarova, Mikhail V. Filippov, Konstantin A. Shestibratov

**Affiliations:** ^1^Vavilov Institute of General Genetics, Russian Academy of Sciences, Gubkin Street 3, Moscow 119991, Russia; ^2^Shemyakin & Ovchinnikov Institute of Bioorganic Chemistry, Russian Academy of Sciences, Pushchino Branch, Pushchino 142290, Russia

## Abstract

Testing systems for molecular identification of micropropagated elite aspen (*Populus tremula* L.) genotypes were developed on the base on microsatellite (SSR) loci. Out of 33 tested microsatellite loci, 14 were selected due to sustainable PCR amplification and substantial variability in elite clones of aspen aimed for establishment of fast-rotated forest plantations. All eight tested clones had different multilocus genotypes. Among 114 trees from three reference native stands located near the established plantations, 80 haplotypes were identified while some repeated genotypes were attributed to natural clones which appeared as a result of sprouting. The selected set of SSR markers showed reliable individual identification with low probability of appearance of identical aspen genotypes (a minimum of 4.8 · 10^−10^ and 1 × 10^−4^ for unrelated and related individuals, resp.). Case studies demonstrating practical applications of the test system are described including analysis of clonal structure and levels of genetic diversity in three natural aspen stands growing in the regions where plantations made of elite clones were established.

## 1. Introduction

The continued degradation of native forests worldwide due to the overexploitation requires introduction of intensive forms of forestry that would favor not only economic effect but also conservation of woody plant genetic resources. This task declares obtaining and maintenance of elite genotypes of forest trees aimed for fast-rotated forest plantations. New highly productive and sustainable to abiotic factors, pests and pathogens cultivars and varieties appear as a result of breeding, selection of mutations, and/or genetic engineering. The clonal propagation of such outstanding individuals ensures fixation of their useful traits both for further breeding experiments and for establishment of targeted forest plantations. In particular, microclonal propagation via* in vitro* culture allows for rapid, large-scale, and cost-effective cloning of individuals with desired traits especially when traditional budding or grafting is impossible or requires special efforts [1–3].

Since morphologically and anatomically different plant clones may look similar, it is essential to reliably identify those including discrimination from each other and from conspecific individuals or representatives of other closely related species. In forestry, the problem of individual identification is especially crucial since the external look of trees is highly dependable on environmental parameters [4]. At particular stages of natural ontogenesis of forest trees, for example, seedlings and saplings, and especially as calluses or explants* in vitro* cell culture, individuals may be indiscernible not only individually but also as far as species diagnoses are concerned. Long generation time and ontogenetically late maturation makes the task of genetic passportization of elite clones in woody plants real. The complex multistage process of obtaining, propagation, and introduction of elite cultivars by breeding or genetic engineering increases the probability of various errors.

Molecular genetic markers (MGM) proved to be very efficient tools for individual identification. Among different MGM classes microsatellites or simple sequence repeats (SSR) fit best to requirements of testing systems for identification due to their specificity, codominance, selective neutrality, sufficient allelic richness, and heterozygosity caused by high mutation rate. Moreover, due to relative genome conservatism within genera and families of plants, SSR-markers and PCR primers for their amplification can be transferrable from one taxon to another.

In several poplar species, the successful DNA fingerprinting and differentiation of clones, cultivars, and varieties were demonstrated by molecular markers including SSR loci [5–9].

In this paper we report on the development of SSR-based testing system for molecular genetic identification of elite micropropagated genotypes of aspen,* Populus tremula* L., aimed at the establishment of fast-growing target forest plantations in several regions of Russian Federation and present the results of its application for individual genotypes discrimination, studies of clonal structure in natural aspen stands, and estimation of genetic variability in populations.

## 2. Material and Methods

The development of the testing system for the identification of elite genotypes comprised the selection of a specific marker set fitting the requirements of high-resolution discrimination of clones and testing its reliability and identification power on a set of elite genotypes and a sufficient number of representatives of a studied species.

### 2.1. Plant Material

Elite aspen and hybrid clones used for development of testing systems for molecular identification have been obtained from micropropagated cell cultures [10] maintained in the Pushchino branch of Shemyakin and Ovchinnikov Institute of Bioorganic Chemistry, Russian Academy of Sciences (Pushchino, Russia). Origin and short description of eight clones are presented in Table 1.

**Figure 1:**
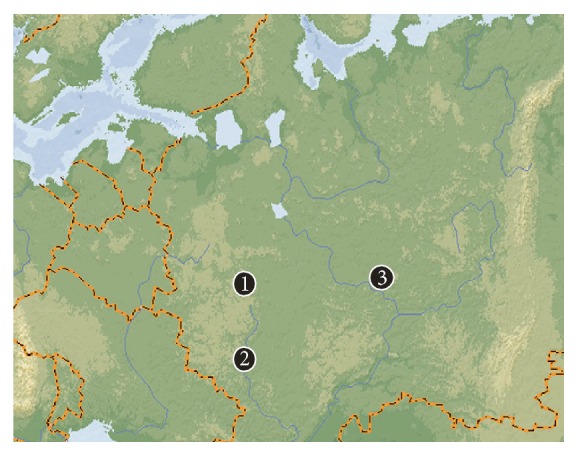
Map of location of plantations made of elite clones and corresponding native aspen stands used as reference populations. (1) Prisady, (2) Voronezh, and (3) Yoshkar-Ola.

Experimental aspen clonal plantations derived from these elite genotypes were established in four regions of European part of Russia (Figure 1). Native aspen stands located closely to these plots were used for studies of clonal structure, evaluation of frequencies of multilocus genotypes, and calculations of probabilities of appearance of identical allelic combinations in a single genotype.

*Prisady*. Experimental plot located 1 km westward of settlement Prisady in Serpukhovsky Raion (district) of Moscow oblast' (Russia). Leaves of 52 young or medium-aged (approximately 10–25 y.o.) aspen trees from a natural multiaged stand located in close proximity (1 km) to this plot have been used as a reference population.

*Voronezh*. Seventeen trees were sampled in a stand adjacent to the experimental plot established in Voronezh Oblast. Additional 13 trees were collected along the bank of Voronezh River within the city of Voronezh close to the plantation.

*Yoshkar-Ola*. Young native stand of aspen was the source of trees used as a reference population for an experimental plot located near the city of Yoshkar-Ola, Republic of Mari-El. Leaves from 32 trees were collected.

In order to minimize the occasional sampling of individuals having vegetative origin (through sprouting) we collected leaves from trees at a distance not less than 15–20 m from each other. This approach was employed for inclusion into reference samples of predominantly open-pollinated seedlings having maximal genetic diversity. However, based exclusively on external look of the trees and distance among them, sampling of the ramets appearing as a result of sprouting could not be avoided, and subsequent genetic analysis confirmed this.

Shoots with leaves were cut off by means of mechanical cutter with an aluminium telescopic mast. Collected shoots with leaves were placed into plastic bags for no more than 6 days at +4°С until processing. Sample preparation included DNA extraction and placement of reserve leaf tissue fragments into labeled zip-bags with silica gel for long-term storage. Outside the period of active vegetation, dormant vegetative buds can be successfully used for DNA extraction.

### 2.2. DNA Extraction

Fragments of leaves approximately 350–500 mg taken from explants growing* in vitro* on a special media (REF) in Petri dishes were used for DNA extraction.

For trees from native populations, we extracted DNA from 350–500 mg fragments of fresh or 200–300 mg of silica-dried leaf tissues by a modified cethyltrimethylammonium bromide (CTAB) method [13, 14].

### 2.3. Microsatellite Analysis

Microsatellites, or SSR, represent a class of tandemly repeated DNA sequences with short (1–6 pairs of nucleotides, bp) motifs differing in copy numbers among individuals due to high mutation rate. Multiple alleles usually found in codominant microsatellite loci create a great variety of unique genotypic combinations which ensures their reliable identification, especially when a sufficient number of loci are employed. Technically, the analysis of SSR polymorphism requires only Polymerase Chain Reaction (PCR) and subsequent electrophoresis (gel or capillary) for fragment analysis. For the development of the testing system we chose the variant of the method that utilizes only basic equipment and simple reagents. This ensured increased reproducibility of the procedures in any PCR laboratory and allowed achieving high cost-effectiveness of the analysis which is crucial for large-scale practical clone identification.

Since the substantial number of nuclear SSR loci for poplars was found in the literature and primer databases we decided to select from several publications and test primers that can be used for routine genotype identification based on very simple equipment without using of DNA-analyzers. An initial set of SSR primers for their potential use as elements of the testing system of molecular identification in aspen was a result of search in bibliographical databases (Thomson Reuters Web of Science, http://webofknowledge.com/) and in Molecular Ecology Resources Primer Database (http://tomato.bio.trinity.edu/).

DNA amplification was performed using PCRCore kits (Isogen Laboratories, Ltd., Moscow, Russia) in BioRad Inc. (USA) Dyad Thermo cycler. Microsatellite loci (listed in Table 2) from series ORPM [15] and WPMS [16] were amplified with specific primers at concentration of 1 mmol/mL and 5–10 ng of target DNA using the following temperature profile: (1) initial denaturation at 94°С for 3 min; (2) 30 cycles consisting of 30 sec of denaturation at 94°С, primer annealing at variable temperature and time (see Table 3 for final annealing temperature recommended after procedure adjustment), and elongation at 72°С for 1.5 min followed by (3) final elongation at 72°С for 10 min and (4) chilling the PCR mixture to 4°С.

PCR products were subjected to electrophoresis in 6% polyacrylamide gel blocks using Tris-EDTA-borate buffer system. After electrophoresis gels were stained in ethidium bromide solution and visualized in UV-light, graphic images were captured and saved using Doc-Print II Vilber Lourmat gel documentation system and processed in graphical editors. Fragment size was estimated by means of specialized software (Photo-Capt). DNA of* E. coli* plasmid pBR322, restricted by endonuclease* Hpa*II was used as a molecular weight marker.

### 2.4. Statistical Analysis

Clone identity was determined using multilocus matches analysis for codominant data. Genotype probability (GP), meaning probability of appearance of particular multilocus combination in population, and probability of identity, estimating probability of random matching of two unrelated (PI) or related (PIsib) individuals by particular set of loci, were calculated based on distribution of allele frequencies in population samples.

Correspondence of observed genotype distributions for each SSR locus to the expected according Hardy-Weinberg equilibrium was tested by chi-square criterion. Allele number and observed and expected heterozygosities were calculated for each native sample. We employed Wright's* F*-statistics for assessment of genetic subdivision among the studied population samples. All the above-mentioned calculations were performed in the add-in for MS Excel, GenAlEx 6.5 [17, 18].

## 3. Results and Discussion

### 3.1. Development of SSR-Based Testing Systems for Genotype Identification in Aspen

For initial testing, we selected 33 heterological tri-, tetra-, penta-, and hexanucleotide microsatellite loci from two sets; series ORPM was designed first for* Populus trichocarpa* [15] and series WPMS was designed for* Populus nigra* [16]. Characteristics of these loci are given in Table 2. Initial testing was done on DNA samples from three clones (47-1, PtV22 и  С-control) from an* in vitro* collection stored and propagated in the Pushchino branch of Shemyakin and Ovchinnikov Institute of Bioorganic Chemistry, Russian Academy of Sciences (Pushchino, Moscow Oblast, Russia). At this stage, their variability was also tested on 20–24 specimens of wild aspen from Novosibirsk Oblast (Western Siberia, Russia) and Krasnoyarsk Krai (Middle Siberia, Russia).

**Figure 2:**
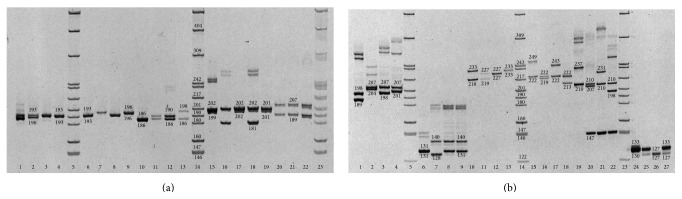
(a) Electrophoretic patterns of PCR-amplified SSR loci* ORPM202*,* ORPM206*,* ORPM220*,* ORPM296,* and* ORPM312* in aspen. Loci: lanes 1–4:* ORPM202*; lanes 6–9:* ORPM206*; lanes 10–13:* ORPM220*; samples: 1, 6, 10, 15, and 19, aspen from native population; 2, 7, 11, 16, and 20, clone С-control; 3, 8, 12, 18, and 21, clone 47-1; 4, 9, 13, 18, and 22, clone PtV22. Lanes 5, 14, and 23: DNA molecular weight marker (DNA of* E. coli* plasmid pBR322, digested by restriction endonuclease* Hpa*II). (b) Electrophoretic patterns of PCR-amplified SSR loci* WPMS15*,* WPMS17*,* WPMS18*,* WPMS19*,* WPMS21*, and* WPMS22* in aspen. Loci: lanes 1–4:* WPMS15*, lanes 6–9:* WPMS17*, lanes 10–13:* WPMS18*, lanes 15–18:* WPMS19*, lanes 19–22:* WPMS21*, and lanes 24–27:* WPMS22.* Samples: 1, 6, 10, 15, 19, and 24, aspen from natural population; 2, 7, 11, 16, 20, and 25, clone С-control; 3, 8, 12, 16, 21, and 26, clone 47-1; 4, 9, 13, 18, 22, and 27, clone PtV22. Lanes 5, 14, and 23, DNA molecular weight marker (DNA of* E. coli* plasmid pBR322, digested by restriction endonuclease* Hpa*II).

As a result of the first phase of testing, 24 loci were successfully amplified and nine other loci failed to produce PCR products. Out of 24 loci that produced PCR fragments, 20 loci were shown to be variable with a number of alleles from two to nine, while four loci that have been successfully amplified were monomorphic (Table 3). After additional testing at variable PCR regimes we finally selected 14 loci with reliable amplification and substantial polymorphism level for inclusion into the test system for molecular genetic identification. Examples of the variability of the selected microsatellite loci in aspen are shown on Figures 2(a) and 2(b).

**Figure 3:**
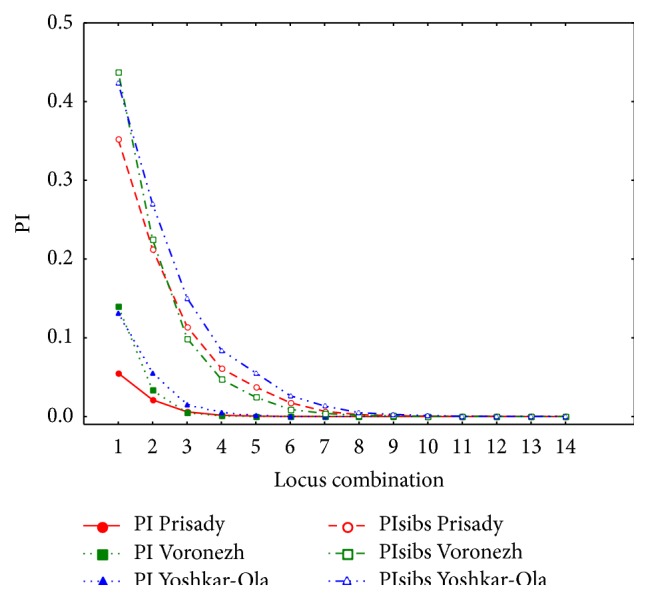
Relationship of PI and PIsibs from the number of used loci. 1 represents locus 1, 2 represents loci 1 + 2, and so forth.

The obtained multilocus genotypes of eight elite clones and reference genotypes of wild trees from native stands are listed in Table S1 in Supplementary Material available online at http://dx.doi.org/10.1155/2015/261518. We analyzed up to 8 ramets sampled at different phases of the microclonal propagation. Within clones, genotypes were stable and unambiguously reproduced among ramets independently of the stage of propagation. After exclusion of ramets of the same genet, both elite clones and aspen genotypes from native populations included in the reference samples were 100% different. Probability of appearance of genotypes (GP: genotype probability) among elite clones varied from 2.4 · 10^−21^ to 1.7 · 10^−11^, among trees in a reference samples from 4.0 · 10^−24^ to 5.8 · 10^−9^. Probability of the occasional coincidence of two unrelated genotypes (PI: probability of identity) varied from 4.8 · 10^−10^ in Yoshkar-Ola to 4.3 · 10^−13^ in Prisady. Adjusted to the theoretical probability of descendance of the compared individuals from the same ancestors more conservative estimate (PIsibs) was within the range of 1.0 · 10^−4^ in Yoshkar-Ola to 9.3 · 10^−6^ in Voronezh. All values are quite low, so that the theoretical frequency of appearance of repeatable genotypes due to recombination of different gametes in course of seed reproduction was not exceeding about 1 accidentally found identical allele combination out of 10000 comparisons. The relationship of PI and PIsibs from the number of loci used is shown at Figure 3 and demonstrates practically negligible probability of appearance of identical genotypes while using first 7 to 8 loci selected for the inclusion into test system. Using all 14 loci ensures additional reliability and robustness of the procedure.

Development of microsatellite loci for species of the genus* Populus* started in late 20th century along with technologies of molecular genetic markers. In 1998, one of the first sets of primers for amplification of dinucleotide SSR loci was designed for American trembling poplar,* Populus tremuloides* [19]. In this paper a successful cross-amplification of the same markers for several other poplar species,* P*.* deltoides*,* P*.* nigra*,* P.* x* canadensis*, and* P. maximowiczii,* was demonstrated. The authors also postulated the broad spectrum of applications of SSR loci for different purposes including clone identification, analysis of the controlled matings, genome mapping, marker-assisted selection, genetic diversity assays, and support of the programs for conservation and sustainable management of forest genetic resources. Subsequent studies provided eight other dinucleotide SSR loci for* Populus tremuloides* [20]. Since that time, microsatellite loci were developed for other species including* P. nigra* [16, 21]. Some of these loci were also amplified in* P. deltoides*,* P. trichocarpa*,* P. tremula*,* P. tremuloides*,* P. candicans*, and* P. lasiocarpa*. Specific SSR loci primers were designed for* Populus euphratica* [22–25]. Later on, this set was used for development of multiplex panels used for genetic diversity estimation in this species [26, 27].

Next generation sequencing was the most efficient way of detection of tandem repeats in poplar genome and design on their base transferrable SSR primers as it was demonstrated in case of balsamic poplar,* Populus trichocarpa* [15, 28]. Such universal cross-amplified loci are used for species and hybrid identification and for estimation of genetic differentiation among congeneric species [5, 9, 29–31].

Microsatellites are also useful for checking of somaclonal diversity within a pool of ramets obtained by microclonal propagation from a single donor tree [5], for identification of individual clones in an aspect of their tolerance to environmental factors [32]. We did not observe any variation in SSR patterns among ramets of the same elite clonal lineage. Nuclear microsatellites along with other classes of molecular markers are employed also for determination of ploidy level in poplars [33, 34], and indications of triploidy were found by us with respect to several genotypes. Triploid aspen hybrids often demonstrate increased vigor and resistance to pathogens, so this matter should be further studied by means of karyological and floating cytometry analysis.

### 3.2. Analysis of Clonal Structure in Native Aspen Stands

Since the very moment of the field sampling of material in wild stands we tried to avoid inclusion of clonal ramets arised as sprouts which is common for aspen. In all studied stands the same sampling scheme was applied keeping at least 15–20 m between the trees. Nevertheless, ramets of the same clone indicating common occurrence of vegetative propagations were found in all of the studied natural populations (Table S1).

*Prisady*. Among the studied 52 trees, we found 41 different haplotypes; one multilocus genotype was found nine times and one was found three times. We concluded that these repeated multilocus combinations resulted from sampling sprouts being ramets of the same clone.

*Voronezh*. Among 17 trees collected at close proximity to the site of the experimental plantation no clonal individuals were found. Thirteen trees collected at the Voronezh River bank were represented by eight genotypes: four of them were in one replicate, three were found to be two ramets of the same clone, and one genotype was found in three copies evidently originated from sprouting of ever existing progenitors. In total, we identified 25 different haplotypes in this reference population sample.

*Yoshkar-Ola*. Thirty-two analyzed trees combined into 13 different haplotypes identified by multilocus matches analysis; one genotype appeared twice, one genotype appeared five times, one genotype appeared seven times, and one genotype appeared nine times. Repeated genotypes evidently corresponded to ramets resulting from sprouting.

We concluded that sprouting and high level of clonality are a widespread phenomenon in native aspen stands. In aspen, as well as in many other poplars, vegetative clones are able to occupy large areas. Therefore, for the collection of a sample free of repeated clonal genotypes distances between trees should be increased up to at least 40–50 m. However, more precise estimation of maximal spread of a single clone over stand territory also requires special exploration.

Among other applications, nuclear SSR loci were useful for the studies of clonal structure and genetic relationships among clone genotypes in native stands [35–38] or between native and artificial stands [39, 40]. Variation in level of clonality was observed, for instance, in black poplar,* P. nigra* [41]. Extensive clonal assemblies were found by means of SSR analysis also in European black poplar [42], in the taxonomic continuum* P. alba *–* P.* x* canescens* on the Iberian Peninsula [43], and in other poplar species.

### 3.3. Levels of Intra- and Interpopulation Genetic Variability

All the loci of the selected set were polymorphic in all studied native population samples. Values of average allele number, effective allele number, and observed and expected heterozygosity were slightly higher in* Prisady* and* Voronezh* than in* Yoshkar-Ola* (Table 4).

The averaged over loci *F*_ST_ (proportion of inter-population variation in total variation, Table 5) was 0.058 ± 0.014 with the highest values observed in two loci: ORPM202 (0.164) and WPMS14 (0.162). AMOVA test showed that 6% of total molecular variation was among populations (significant, *P* = 0.001), 10% were among individuals, and 84% were within individuals.

Microsatellites isolated from nuclear as well as chloroplast genomes were employed for the analysis of genetic diversity and genetic structure in various poplar species [42, 44–60]. Levels of intrapopulation genetic diversity observed by us in native aspen stands were within the range of values commonly observed in poplars and estimated by SSR loci analysis ([47] and references therein).

A substantial number of studies employing microsatellite technique were focused on the detection of hybridization in natural [31, 46, 61–63] or artificial [64] stands of poplars. SSR loci help to reveal mechanisms of interspecific crossing and reproductive isolation [65–67]. In this study we concentrated on among-individual and among-population variation rather than on interspecific differences, but with respect to some of the studied elite clones their hybrid origin should be genetically tested using a complex set of markers but of nuclear and cytoplasmic localization. Hi-fidelity identification of interspecific hybrids and industrial clones was reported in a considerable number of publications [5, 6, 51, 68–74]. A remarkable publication reports on the genetic identity of some industrial clones of poplars previously treated as different [68, 69].

The practical task of identity monitoring of commercial clones in* in vitro* collections [75, 76] was similar to that employed in the present research. In all applications where reliable individual identification was required SSRs showed high effectiveness.

## 4. Conclusion

Application of nuclear microsatellite loci for genetic identification and assessment of genetic variability in aspen elite clones and native stands showed high effectiveness of the developed low-cost SSR-based testing system. Reliable authentication of clones ensures genetic monitoring of microclonal propagation and allows revealing clonality in native stands. We demonstrated that the same set of microsatellite loci can be successfully employed for estimation of levels of intra- and interpopulation genetic variability in aspen. Reconstruction of kinship among individual elite clones or genetic relationships of naturally mating populations are perspective tasks that can be realized in the future using the same markers.

## Supplementary Material

Suppplementary Table S1 contains a matrix of genotypes of aspen elite clones and trees from three native reference stands by 14 microsatellite loci, probability of appearance of each genotype in a sample and number of ramets of individual clones found in natural populations.

## Figures and Tables

**Table 1:** Elite aspen and hybrid clones and their characteristics.

Original genotype	Putative species/hybrid identity	Origin	Description	Clones and clonal lineages obtained based on original genotypes
PtV22	Putatively *Populus tremula*	Minsk Oblast, Belarus, breeding form obtained in Institute of Forest, National Academy of Belarus, Gomel, Belarus, provided by V. E. Padutov	Diploid green-bark aspen form. Characterized by fast growth and resistance to heart rot caused by pathogen fungus *Phellinus tremula*	Ptv22-1, Ptv22-2, Ptv22-3, 21mut, 2mut, 14mut, 4mut, 12mut

Pt	*P. tremula*	Leningrad Oblast, Russia, breeding form obtained in St. Petersburg Research Institute for Forestry, provided by D. A. Shabunin	Diploid giant aspen form. Characterized by fast growth and resistance to heart rot caused by pathogen fungus *Phellinus tremula*	Pt2, Pt3

F2	*P. tremula*	Kostroma Oblast, Russia, breeding form obtained by S. N. Bagayev, provided by D. A. Shabunin	Diploid female clone. Highly productive (plus 51% by sum of stem cross section squares and plus 43% by growing stock). Increased wood density of 475 kg/m^3^[11]	F2-1, F2-2, F2-3

47	*P. tremula*	Latvian State Forest Research Institute “Silava,” Latvia, provided by Dr. Arnis Gailis	Diploid aspen form. Productivity of 180–200 m^3^/haat age 12 under density of 1100 stems/ha. Characterized by resistance to heart rot caused by pathogen fungus *Phellinus tremula *[12]	47-1, 47-1-1-31, 47-1-1-22, 47-1-2-27, 47-1-1-19, 47-1-2-53

С-control	*P. tremuloides × P. tremula*	-“-	Diploid hybrid form. Productivity of 180–200 m^3^/ha at age 12 under density of 1100 stems/ha [12]	С-1, С-2, С-3

23	*P. tremuloides × P. tremula*	-“-	Diploid hybrid form. Maximal productivity of 200–250 m^3^/ha demonstrated at age 12 under density of 1100 stems/ha [12]. High wood density	L23-1, L23-2, L23-3

4	*P. tremuloides × P. tremula*	-“-	Maximal productivity of 250–300 m^3^/ha demonstrated at age 12 under density of 2500 stems/ha [12]	L4-1, L4-2, L4-3

No. 3-understory	*P. tremula*	Republic of Tatarstan, breeding form by A. H. Gaziulllin, provided by N. R. Garipov	Triploid aspen form. Characterized by fast growth and resistance to heart rot caused by pathogen fungus *Phellinus tremula*	No. 3-understory-1

**Table 2:** Microsatellite loci tested for PCR amplification in aspen.

Loci	Primer sequences (5′- 3′)	Repeat motif	Fragment size (bp)^3^
ORPM14^1^	F- GGGCTGCAGCAGATATTGA R- CCAAAGGAACCCAAAGAAGA	(GCTC)_4_	146–162

ORPM18^1^	F- AGCAGAGATCGATGCTGAGG R- AATTTCTCGCTTCTCGCATT	(TTTA)_4_	205

ORPM36^1^	F- AGCCTCCAAACACCATGAAC R- ACAGTGGTGTGGATCCTGCT	(GAAA)_4_	213

ORPM60^1^	F- ATAGCGCCAGAAGCAAAAAC R- AAGCAGAAAGTCGTAGGTTCG	(AAT)_5_	212

ORPM79^1^	F- GAAGCTGAAAACAACAACAAACA R- GGGTTTTTAACATAATAAAAGCTTGG	(AAT)_4_	160

ORPM81^1^	F- GCTGCAGCCAAACAAAGC R- CAGAAATCCCACCCAAACC	(TATT)_4_	142–158

ORPM84^1^	F- CTGCAGCCTTACCACCATTT R- CGTGTTCGAGATTGGGATTT	(AAG)_4_	171

ORPM86^1^	F- CCACATCCATAGCTCTGCAAC R- GTACTACCTCGCCTGCCAAC	(CTT)_5_	204

ORPM107^1^	F- AATCTGGTGGCTTGCCTCT R- TTGAGGAACACGTGCAGACT	(TAAA)_4_	190

ORPM117^1^	F- CCCCCTAATTACCTTGGAAAC R- TTGTTTGTATCTCCTCCGTTGA	(ATTA)_4_	210

ORPM158^1^	F- GCTGAAACATCCTTCATGGTC R- CGAAGCTGCATAAGCATCAA	(TTTC)_4_	200

ORPM193^1^	F- CCGCTGGATTTGTTTGTTTT R- TGAGCAGAAAGATGCGAAGA	(ATTTT)_4_	187–207

ORPM202^1^	F- TCGCAAAAGATTCTCCCAGT R- TTCAAATCCCGGTAATGCTC	(TAA)_5_	184–190

ORPM206^1^	F- CCGTGGCCATTGACTCTTTA R- GAACCCATTTGGTGCAAGAT	(GCT)_7_	190–208

ORPM220^1^	F- AGCTAGCCTGTCGTCAAGGA R- CAAGGAAGCATTCTCGCAAT	(TTTA)_6_	178–222

ORPM296^1^	F- CGAAGCCATTGACCCAGTAT R- GGGCATCTCTCTCCTTTCAA	(GTTCTG)_4_	199

ORPM312^1^	F- GTGGGGATCAATCCAAAAGA R- CCCATATCAAACCATTTGAAAAA	(CCT)_6_	194

ORPM366^1^	F- CCTTGAGGGGACACTTCGAT R- AAAGAGTTGAGCCCTTGGTG	(TTA)_5_	156

ORPM371^1^	F- CCGGACTCTCACAAATCTCC R- TGCTTTTGCTCCTGGTTCTT	(TCTT)_6_	192–200

ORPM372^1^	F- AGCTCTTCTGCTGGTGCTGT R- GAGGGAGGGAGGGTAAAAGA	(TCTT)_5_	190

ORPM415^1^	F- CTCGGTGCAAATATCGGTTC R- AGATCGATGGTCCTTTCCTG	(GGCG)_4_	225

ORPM484^1^	F- CAAAATGGCAATCCAAGGTT R- CCAAGCTTCCAATTGAGTCC	(TTAA)_4_	190–206

ORPM488^1^	F- CTCCAGCCGCTTCTATCCTT R- TGTCGTGGGAAAGAACCAGT	(TTA)_6_	200

ORPM496^1^	F- CAGCAGTGCAAGCTCCTAAA R- GGCCACTGACAGAGACCAAG	(GGA)_4_	185

WPMS14^2^	F- CAGCCGCAGCCACTGAGAAATC R- GCCTGCTGAGAAGACTGCCTTGAC	(CGT)_28_	215–287

WPMS15^2^	F- CAACAAACCATCAATGAAGAAGAC R- AGAGGGTGTTGGGGGTGACTA	(CCT)_14_	201–219

WPMS16^2^	F- CTCGTACTATTTCCGATGATGACC R- AGATTATTAGGTGGGCCAAGGACT	(GTC)_8_	139–184

WPMS17^2^	F- ACATCCGCCAATGCTTCGGTGTTT R- GTGACGGTGGTGGCGGATTTTCTT	(CAC)_15_	122–146

WPMS18^2^	F- CTTCACATAGGACATAGCAGCATC R- CACCAGAGTCATCACCAGTTATTG	(GTG)_13_	219–248

WPMS19^2^	F- AGCCACAGCAAATTCAGATGATGC R- CCTGCTGAGAAGACTGCCTTGACA	(CAG)_28_	180–234

WPMS20^2^	F- GTGCGCACATCTATGACTATCG R- ATCTTGTAATTCTCCGGGCATCT	(TTCTGG)_8_	210–222

WPMS21^2^	F- TGCTGATGCAAAAGATTTAG R- TTGGAACTTCAACATTCAGAT	(GCT)_45_	287–326

WPMS22^2^	F- ACATGCTACGTGTTTGGAATG R- ATCGTATGGATGTAATTGTCTTA	(TGA)_23_	100–135

Comments: ^1^Tuskan et al., 2004 [15];^ 2^Smulders et al., 2001 [16]; ^3^by literature data in different *Populus* species (*P. tremula*, *P.* x* canescens*, and *P. alba*).

**Table 3:** Results of testing of microsatellite loci in aspen.

Locus	PCR amplification	Fragment size range (bp)	Number of alleles	Status^1^	Included in testing system
ORPM14	Yes	146–162	4	P	No
ORPM18	No	—	—	N	No
ORPM36	Yes	217	1	M	No
ORPM60	No	—	—	N	No
ORPM79	No	—	—	N	No
ORPM81	No	—	—	N	No
ORPM84	No	—	—	N	No
ORPM86	Yes	204–216	4	P	No
ORPM107	No	—	—	N	No
ORPM117	Yes	218	1	M	No
ORPM158	Yes	200	1	M	No
ORPM193	Yes	182–207	6	P	Yes
ORPM202	Yes	184–193	5	P	Yes
ORPM206	Yes	190–196	3	P	Yes
ORPM220	Yes	178–198	5	P	Yes
ORPM296	Yes	201–183	4	P	Yes
ORPM312	Yes	189–201	4	P	No
ORPM366	No	—	—	N	No
ORPM371	Yes	192–200	3	P	No
ORPM372	No	—	—	N	No
ORPM415	Yes	~280	1	M	No
ORPM484	Yes	190–206	3	P	No
ORPM488	Yes	197–203	2	P	No
ORPM496	No	—	—	N	No
WPMS14	Yes	224–243	3	P	Yes
WPMS15	Yes	189–207	5	P	Yes
WPMS16	Yes	139–184	9	P	Yes
WPMS17	Yes	122–146	7	P	Yes
WPMS18	Yes	219–248	7	P	Yes
WPMS19	Yes	210–252	9	P	Yes
WPMS20	Yes	210–222	4	P	Yes
WPMS21	Yes	196–240	5	P	Yes
WPMS22	Yes	115–135	3	P	Yes

^1^P: polymorphic, M: monomorphic, and N: no PCR amplification.

**Table 4:** Parameters of genetic variability in aspen populations.

Population		*N*	*N*_*a*_	*N*_*e*_	*H*_*O*_	*H*_*E*_	*UH*_*E*_	*F*
Prisady	Mean	41	6.429	3.921	0.631	0.661	0.669	0.047
	s.e.		0.850	0.602	0.059	0.044	0.045	0.065
Voronezh	Mean	25	7.429	3.970	0.580	0.687	0.701	0.188
	s.e.		1.274	0.584	0.068	0.035	0.036	0.076
Yoshkar-Ola	Mean	13	4.643	2.738	0.522	0.567	0.590	0.109
	s.e.		0.541	0.343	0.072	0.043	0.045	0.087
Total mean	Mean	26.333	6.167	3.543	0.578	0.639	0.654	0.115
	s.e.	1.791	0.558	0.308	0.038	0.024	0.025	0.044

s.e.: standard error.

*N*: sample size.

*N*_*a*_: mean number of alleles.

*N*_*e*_: effective number of alleles.

*H*_*O*_: observed heterozygosity.

*H*_*E*_: expected heterozygosity.

*UH*_*E*_: unbiased expected heterozygosity.

*F*: fixation index (intrapopulation coefficient of inbreeding).

**Table 5:** *F*-statistics for three natural aspen populations.

Locus	*F*_IS_	*F*_IT_	*F*_ST_
*ORPM193*	0.158	0.230	0.086
*ORPM202*	0.514	0.593	0.164
*ORPM206*	0.276	0.363	0.120
*ORPM220*	0.066	0.096	0.032
*ORPM296*	0.046	0.067	0.023
*WPMS14*	0.074	0.224	0.162
*WPMS15*	−0.187	−0.144	0.036
*WPMS16*	−0.105	−0.083	0.019
*WPMS17*	−0.066	−0.028	0.036
*WPMS18*	0.305	0.322	0.025
*WPMS19*	−0.034	−0.003	0.029
*WPMS20*	0.111	0.131	0.023
*WPMS21*	−0.057	−0.027	0.028
*WPMS22*	0.563	0.578	0.035

Mean	0.119	0.166	0.058
s.e.	0.060	0.062	0.014

s.e.: standard error.
