# Supplementary material for: Application of Microsatellite Loci for Molecular Identification of Elite Genotypes, Analysis of Clonality, and Genetic Diversity in Aspen Populus tremula L. (Salicaceae)
Source: Int J Plant Genomics. 2015 Dec 28;2015:261518. doi: 10.1155/2015/261518 (PMC4707373; doi:10.1155/2015/261518)
Supplement: Supplementary file 1 — Suppplementary Table S1 contains a matrix of genotypes of aspen elite clones and trees from three native reference stands by 14 microsatellite loci, probability of appearance of each genotype in a sample and number of ramets of individual clones found in natural populations. [file 261518.f1.doc]

Table 1S. Genotypes of aspen elite clones and trees from three native reference stands by 14 microsatellite loci

| Specimen/Loci | GP^1^ | *ORPM193* | | *ORPM202* | | *ORPM206* | | *ORPM220* | | *ORPM296* | | *WPMS14* | | *WPMS15* | | *WPMS16* | | *WPMS17* | | *WPMS18* | | *WPMS19* | | *WPMS20* | | *WPMS21* | | *WPMS22* | | NR^2^ |
| --- | --- | --- | --- | --- | --- | --- | --- | --- | --- | --- | --- | --- | --- | --- | --- | --- | --- | --- | --- | --- | --- | --- | --- | --- | --- | --- | --- | --- | --- | --- |
| elite clones |  |  |  |  |  |  |  |  |  |  |  |  |  |  |  |  |  |  |  |  |  |  |  |  |  |  |  |  |  |  |
| PtrC-control | 2.4•10^-11^ | 197 | 197 | 190 | 187 | 193 | 193 | 190 | 186 | 199 | 181 | 232 | 202 | 207 | 204 | 175 | 157 | 140 | 128 | 227 | 218 | 222 | 219 | 233 | 215 | 210 | 207 | 133 | 133 | 1 |
| Pt47-1 | 3.6•10^-12^ | 197 | 197 | 190 | 190 | 190 | 190 | 190 | 186 | 199 | 199 | 220 | 202 | 207 | 198 | 175 | 157 | 140 | 131 | 227 | 227 | 243 | 222 | 233 | 215 | 231 | 210 | 133 | 127 | 1 |
| PtV22 | 4.1•10^-12^ | 195 | 187 | 190 | 190 | 193 | 193 | 198 | 186 | 199 | 181 | 232 | 202 | 207 | 201 | 181 | 169 | 140 | 131 | 233 | 227 | 222 | 213 | 233 | 215 | 210 | 198 | 133 | 133 | 1 |
| Pt2 | 9.4•10^-11^ | 197 | 197 | 190 | 187 | 193 | 190 | 186 | 178 | 199 | 193 | 223 | 217 | 198 | 189 | 172 | 169 | 125 | 125 | 227 | 227 | 222 | 213 | 233 | 227 | 210 | 198 | 133 | 133 | 1 |
| №3 | 6.4•10^-12^ | 197 | 187 | 190 | 187 | 193 | 190 | 186 | 178 | 199 | 199 | 232 | 214 | 201 | 198 | 169 | 157 | 131 | 125 | 227 | 224 | 225 | 219 | 233 | 233 | 216 | 213 | 133 | 130 | 1 |
| F2-1 | 2.8•10^-11^ | 197 | 197 | 187 | 187 | 193 | 190 | 186 | 178 | 199 | 193 | 223 | 217 | 198 | 189 | 172 | 169 | 125 | 125 | 227 | 227 | 222 | 213 | 233 | 227 | 210 | 198 | 133 | 133 | 1 |
| L4-1 | 1.7•10^-12^ | 197 | 182 | 190 | 190 | 193 | 193 | 190 | 186 | 199 | 181 | 232 | 214 | 201 | 198 | 175 | 169 | 140 | 131 | 233 | 218 | 231 | 222 | 233 | 215 | 219 | 210 | 133 | 127 | 1 |
| L23-1 | 4.3•10^-12^ | 197 | 187 | 190 | 187 | 193 | 193 | 178 | 178 | 199 | 199 | 217 | 211 | 201 | 201 | 157 | 157 | 131 | 128 | 233 | 218 | 225 | 219 | 233 | 233 | 216 | 210 | 133 | 133 | 1 |
| trees from native  stands |  |  |  |  |  |  |  |  |  |  |  |  |  |  |  |  |  |  |  |  |  |  |  |  |  |  |  |  |  |  |
| Prisady1 | 4.4•10^-17^ | 197 | 197 | 190 | 190 | 193 | 193 | 186 | 182 | 199 | 193 | 232 | 202 | 198 | 189 | 184 | 163 | 131 | 131 | 230 | 224 | 219 | 213 | 233 | 227 | 207 | 198 | 0 | 0 | 1 |
| Prisady2 | 2.1•10^-16^ | 195 | 195 | 190 | 190 | 193 | 190 | 186 | 182 | 199 | 199 | 247 | 247 | 204 | 201 | 184 | 157 | 131 | 131 | 233 | 224 | 228 | 228 | 233 | 233 | 243 | 213 | 130 | 130 | 1 |
| Prisady5 | 3.5•10^-15^ | 195 | 187 | 187 | 187 | 190 | 190 | 186 | 178 | 199 | 193 | 232 | 217 | 201 | 198 | 187 | 157 | 131 | 125 | 221 | 221 | 222 | 219 | 233 | 227 | 210 | 207 | 130 | 130 | 1 |
| Prisady6 | 3.9•10^-17^ | 197 | 187 | 190 | 187 | 193 | 190 | 186 | 178 | 199 | 175 | 232 | 223 | 189 | 186 | 187 | 157 | 131 | 131 | 224 | 224 | 195 | 195 | 233 | 209 | 243 | 180 | 133 | 133 | 2 |
| Prisady7 | 5.1•10^-13^ | 187 | 185 | 190 | 187 | 190 | 190 | 186 | 186 | 199 | 193 | 232 | 232 | 201 | 189 | 157 | 157 | 125 | 125 | 230 | 230 | 225 | 219 | 233 | 227 | 213 | 207 | 133 | 133 | 1 |
| Prisady8 | 1.0•10^-13^ | 187 | 185 | 190 | 187 | 193 | 190 | 186 | 182 | 199 | 199 | 247 | 202 | 201 | 201 | 163 | 157 | 131 | 125 | 224 | 224 | 222 | 222 | 233 | 233 | 240 | 210 | 133 | 133 | 1 |
| Prisady9 | 6.1•10^-15^ | 197 | 195 | 187 | 187 | 193 | 193 | 186 | 186 | 199 | 187 | 232 | 223 | 201 | 201 | 187 | 169 | 131 | 125 | 218 | 218 | 213 | 213 | 233 | 221 | 198 | 198 | 133 | 133 | 2 |
| Prisady11 | 2.0•10^-14^ | 187 | 182 | 190 | 187 | 193 | 190 | 178 | 178 | 199 | 199 | 232 | 232 | 201 | 189 | 175 | 169 | 131 | 131 | 230 | 230 | 228 | 213 | 233 | 233 | 216 | 198 | 133 | 133 | 1 |
| Prisady13 | 2.4•10^-15^ | 195 | 182 | 187 | 187 | 193 | 193 | 178 | 178 | 199 | 199 | 232 | 223 | 201 | 189 | 157 | 157 | 131 | 125 | 236 | 230 | 228 | 222 | 233 | 233 | 216 | 210 | 130 | 130 | 1 |
| Prisady15 | 1.1•10^-14^ | 200 | 190 | 190 | 187 | 199 | 190 | 186 | 186 | 199 | 199 | 217 | 205 | 201 | 198 | 163 | 157 | 131 | 125 | 239 | 218 | 222 | 213 | 233 | 233 | 210 | 198 | 133 | 130 | 2 |
| Prisady16 | 6.1•10^-15^ | 200 | 197 | 187 | 187 | 193 | 193 | 186 | 178 | 199 | 199 | 232 | 217 | 204 | 198 | 172 | 169 | 128 | 125 | 230 | 218 | 219 | 213 | 233 | 233 | 207 | 198 | 133 | 130 | 1 |
| Prisady17 | 4.3•10^-16^ | 200 | 195 | 190 | 187 | 193 | 190 | 186 | 178 | 199 | 187 | 217 | 211 | 204 | 201 | 169 | 169 | 131 | 131 | 236 | 236 | 222 | 219 | 233 | 221 | 210 | 207 | 133 | 133 | 3 |
| Prisady19 | 6.9•10^-17^ | 197 | 187 | 190 | 190 | 190 | 190 | 186 | 178 | 199 | 193 | 232 | 214 | 201 | 189 | 187 | 163 | 125 | 125 | 0 | 0 | 228 | 222 | 233 | 227 | 216 | 210 | 133 | 127 | 2 |
| Prisady20 | 1.6•10^-16^ | 190 | 187 | 190 | 187 | 190 | 190 | 186 | 178 | 193 | 187 | 232 | 229 | 201 | 189 | 169 | 163 | 128 | 125 | 230 | 230 | 225 | 225 | 227 | 221 | 243 | 213 | 133 | 133 | 1 |
| Prisady21 | 1.4•10^-12^ | 190 | 185 | 187 | 187 | 193 | 190 | 186 | 178 | 199 | 199 | 232 | 217 | 204 | 198 | 157 | 157 | 131 | 125 | 233 | 224 | 249 | 225 | 233 | 233 | 243 | 216 | 133 | 130 | 1 |
| Prisady22 | 4.6•10^-16^ | 195 | 195 | 190 | 190 | 193 | 190 | 186 | 182 | 199 | 199 | 247 | 247 | 204 | 201 | 184 | 157 | 131 | 131 | 239 | 227 | 249 | 225 | 233 | 233 | 243 | 216 | 130 | 130 | 1 |
| Prisady23 | 3.7•10^-14^ | 197 | 187 | 187 | 187 | 193 | 193 | 186 | 182 | 199 | 199 | 232 | 232 | 204 | 204 | 157 | 157 | 131 | 128 | 224 | 218 | 222 | 210 | 233 | 233 | 210 | 198 | 130 | 130 | 1 |
| Prisady24 | 4.7•10^-14^ | 197 | 185 | 187 | 187 | 193 | 190 | 186 | 178 | 199 | 199 | 217 | 214 | 201 | 198 | 169 | 157 | 131 | 125 | 227 | 227 | 225 | 213 | 233 | 233 | 216 | 201 | 133 | 133 | 1 |
| Prisady25 | 1.3•10^-16^ | 187 | 187 | 190 | 187 | 190 | 190 | 186 | 178 | 199 | 187 | 232 | 232 | 204 | 189 | 169 | 163 | 128 | 125 | 0 | 0 | 249 | 225 | 227 | 221 | 243 | 210 | 0 | 0 | 1 |
| Prisady26 | 3.9•10^-15^ | 197 | 187 | 187 | 187 | 193 | 193 | 186 | 178 | 199 | 199 | 232 | 220 | 207 | 201 | 169 | 157 | 128 | 125 | 239 | 233 | 225 | 195 | 233 | 233 | 210 | 180 | 133 | 133 | 1 |
| Prisady27 | 7.5•10^-14^ | 187 | 187 | 190 | 190 | 193 | 193 | 178 | 178 | 199 | 199 | 232 | 232 | 204 | 201 | 193 | 157 | 131 | 125 | 239 | 227 | 228 | 195 | 233 | 233 | 213 | 180 | 133 | 133 | 1 |
| Prisady28 | 8.1•10^-15^ | 197 | 185 | 190 | 190 | 193 | 190 | 186 | 186 | 199 | 199 | 253 | 217 | 204 | 201 | 163 | 163 | 131 | 125 | 233 | 233 | 225 | 195 | 233 | 233 | 210 | 180 | 133 | 133 | 1 |
| Prisady29 | 1.5•10^-12^ | 197 | 190 | 190 | 187 | 196 | 190 | 186 | 178 | 199 | 199 | 232 | 232 | 204 | 201 | 169 | 163 | 131 | 125 | 227 | 224 | 249 | 219 | 233 | 233 | 240 | 207 | 133 | 133 | 1 |
| Prisady30 | 1.1•10^-13^ | 200 | 190 | 190 | 187 | 193 | 190 | 186 | 178 | 199 | 187 | 232 | 232 | 189 | 189 | 184 | 157 | 131 | 131 | 218 | 218 | 225 | 222 | 233 | 221 | 213 | 207 | 133 | 133 | 1 |
| Prisady31 | 5.5•10^-16^ | 190 | 187 | 187 | 187 | 193 | 190 | 178 | 178 | 199 | 193 | 247 | 217 | 201 | 189 | 187 | 163 | 131 | 125 | 233 | 227 | 234 | 225 | 233 | 227 | 222 | 213 | 133 | 133 | 1 |
| Prisady32 | 1.2•10^-12^ | 190 | 185 | 190 | 187 | 193 | 190 | 178 | 178 | 199 | 199 | 232 | 232 | 201 | 189 | 175 | 169 | 131 | 131 | 227 | 227 | 225 | 210 | 233 | 233 | 213 | 198 | 133 | 133 | 1 |
| Prisady33 | 3.1•10^-15^ | 205 | 190 | 190 | 187 | 199 | 190 | 186 | 186 | 199 | 199 | 217 | 205 | 201 | 198 | 163 | 157 | 131 | 125 | 239 | 218 | 222 | 210 | 233 | 233 | 210 | 198 | 133 | 130 | 1 |
| Prisady34 | 2.6•10^-14^ | 195 | 195 | 190 | 190 | 199 | 193 | 178 | 178 | 199 | 199 | 247 | 232 | 198 | 189 | 181 | 157 | 125 | 125 | 227 | 227 | 219 | 210 | 233 | 233 | 207 | 198 | 133 | 133 | 1 |
| Prisady35 | 1.4•10^-14^ | 197 | 187 | 187 | 187 | 190 | 190 | 186 | 178 | 199 | 193 | 232 | 223 | 198 | 195 | 163 | 157 | 143 | 131 | 233 | 233 | 219 | 210 | 233 | 227 | 207 | 198 | 133 | 130 | 1 |
| Prisady36 | 6.2•10^-14^ | 195 | 190 | 187 | 187 | 193 | 193 | 186 | 178 | 199 | 187 | 247 | 232 | 201 | 198 | 193 | 163 | 125 | 125 | 227 | 224 | 225 | 195 | 233 | 221 | 213 | 180 | 133 | 133 | 1 |
| Prisady39 | 7.2•10^-13^ | 195 | 190 | 190 | 187 | 193 | 190 | 186 | 178 | 199 | 187 | 232 | 232 | 201 | 198 | 193 | 169 | 131 | 125 | 239 | 227 | 222 | 195 | 233 | 221 | 207 | 180 | 133 | 133 | 2 |
| Prisady40 | 2.3•10^-14^ | 190 | 187 | 190 | 187 | 190 | 190 | 186 | 178 | 193 | 187 | 232 | 232 | 201 | 189 | 169 | 163 | 128 | 125 | 227 | 227 | 249 | 222 | 227 | 221 | 243 | 210 | 133 | 133 | 2 |
| Prisady41 | 1.5•10^-11^ | 187 | 185 | 190 | 190 | 193 | 190 | 186 | 182 | 199 | 199 | 232 | 232 | 198 | 189 | 169 | 163 | 131 | 125 | 227 | 224 | 222 | 210 | 233 | 233 | 210 | 198 | 133 | 133 | 1 |
| Prisady43 | 5.9•10^-15^ | 195 | 187 | 187 | 187 | 190 | 190 | 186 | 178 | 199 | 193 | 232 | 223 | 198 | 195 | 163 | 157 | 143 | 131 | 233 | 233 | 219 | 210 | 233 | 227 | 204 | 198 | 133 | 130 | 2 |
| Prisady44 | 1.1•10^-13^ | 197 | 195 | 190 | 187 | 193 | 190 | 186 | 182 | 199 | 199 | 232 | 214 | 198 | 189 | 181 | 157 | 131 | 131 | 233 | 227 | 219 | 210 | 233 | 233 | 204 | 198 | 133 | 133 | 1 |
| Prisady45 | 8.7•10^-13^ | 195 | 187 | 190 | 187 | 196 | 190 | 186 | 178 | 199 | 199 | 232 | 232 | 204 | 198 | 169 | 163 | 131 | 125 | 227 | 224 | 249 | 219 | 233 | 233 | 240 | 204 | 133 | 133 | 1 |
| Prisady46 | 4.6•10^-17^ | 200 | 185 | 187 | 187 | 193 | 190 | 186 | 178 | 199 | 199 | 232 | 232 | 195 | 195 | 208 | 181 | 125 | 125 | 224 | 224 | 222 | 195 | 233 | 233 | 207 | 180 | 0 | 0 | 1 |
| Prisady48 | 3.5•10^-14^ | 195 | 187 | 187 | 187 | 190 | 190 | 186 | 178 | 199 | 193 | 232 | 217 | 198 | 195 | 184 | 157 | 131 | 125 | 224 | 224 | 222 | 219 | 233 | 227 | 207 | 204 | 130 | 130 | 2 |
| Prisady50 | 5.1•10^-15^ | 195 | 195 | 187 | 187 | 193 | 193 | 186 | 186 | 199 | 187 | 232 | 223 | 198 | 195 | 184 | 169 | 131 | 125 | 218 | 218 | 210 | 210 | 233 | 221 | 198 | 198 | 133 | 133 | 2 |
| Prisady51 | 1.9•10^-14^ | 185 | 185 | 190 | 187 | 199 | 190 | 186 | 178 | 199 | 187 | 232 | 232 | 198 | 195 | 184 | 169 | 131 | 125 | 224 | 224 | 249 | 219 | 233 | 221 | 240 | 207 | 133 | 133 | 1 |
| Prisady52 | 8.8•10^-19^ | 185 | 185 | 190 | 187 | 190 | 190 | 186 | 178 | 193 | 187 | 232 | 232 | 198 | 186 | 169 | 163 | 128 | 125 | 0 | 0 | 249 | 225 | 227 | 221 | 240 | 213 | 0 | 0 | 1 |
| Voronezh1 | 2.5•10^-14^ | 190 | 190 | 190 | 190 | 196 | 193 | 178 | 178 | 199 | 187 | 232 | 223 | 201 | 198 | 190 | 169 | 131 | 125 | 227 | 218 | 216 | 210 | 233 | 233 | 207 | 198 | 0 | 0 | 1 |
| Voronezh2 | 5.1•10^-14^ | 190 | 190 | 190 | 190 | 193 | 193 | 186 | 178 | 199 | 187 | 232 | 232 | 198 | 189 | 184 | 169 | 131 | 131 | 227 | 224 | 249 | 195 | 233 | 221 | 243 | 180 | 133 | 133 | 1 |
| Voronezh3 | 1.2•10^-11^ | 200 | 190 | 193 | 190 | 196 | 193 | 186 | 186 | 199 | 199 | 232 | 232 | 198 | 198 | 157 | 157 | 125 | 125 | 227 | 227 | 219 | 216 | 233 | 233 | 207 | 207 | 133 | 133 | 1 |
| Voronezh4 | 2.5•10^-17^ | 190 | 190 | 190 | 190 | 196 | 193 | 190 | 190 | 199 | 193 | 232 | 211 | 201 | 201 | 187 | 169 | 131 | 131 | 239 | 224 | 231 | 222 | 233 | 227 | 222 | 213 | 133 | 133 | 1 |
| Voronezh5 | 5.3•10^-13^ | 200 | 200 | 190 | 190 | 196 | 193 | 178 | 178 | 199 | 187 | 247 | 232 | 204 | 189 | 169 | 157 | 125 | 125 | 227 | 224 | 249 | 219 | 233 | 233 | 243 | 210 | 133 | 133 | 1 |
| Voronezh6 | 1.3•10^-14^ | 190 | 195 | 190 | 190 | 196 | 196 | 186 | 186 | 187 | 187 | 220 | 217 | 198 | 198 | 169 | 157 | 131 | 125 | 227 | 218 | 219 | 216 | 221 | 221 | 210 | 207 | 133 | 133 | 1 |
| Voronezh7 | 6.6•10^-17^ | 190 | 190 | 193 | 193 | 196 | 196 | 186 | 182 | 199 | 193 | 241 | 217 | 198 | 198 | 169 | 157 | 131 | 125 | 239 | 218 | 228 | 222 | 233 | 227 | 219 | 213 | 133 | 133 | 1 |
| Voronezh8 | 1.0•10^-21^ | 197 | 202 | 193 | 193 | 202 | 196 | 182 | 178 | 193 | 193 | 232 | 205 | 204 | 198 | 208 | 169 | 131 | 125 | 227 | 227 | 246 | 231 | 227 | 227 | 240 | 222 | 133 | 133 | 1 |
| Voronezh9 | 3.4•10^-14^ | 190 | 190 | 193 | 193 | 196 | 193 | 186 | 182 | 199 | 199 | 247 | 217 | 201 | 189 | 163 | 157 | 131 | 125 | 233 | 224 | 219 | 210 | 233 | 233 | 210 | 198 | 133 | 133 | 1 |
| Voronezh10 | 8.0•10^-14^ | 195 | 190 | 190 | 190 | 196 | 193 | 186 | 178 | 199 | 199 | 232 | 220 | 201 | 198 | 187 | 163 | 143 | 131 | 233 | 227 | 222 | 219 | 233 | 233 | 213 | 210 | 133 | 133 | 1 |
| Voronezh11 | 5.8•10^-13^ | 200 | 190 | 193 | 190 | 193 | 193 | 186 | 186 | 199 | 199 | 232 | 223 | 201 | 198 | 187 | 172 | 143 | 125 | 233 | 233 | 219 | 216 | 233 | 233 | 210 | 207 | 133 | 133 | 1 |
| Voronezh12 | 1.3•10^-15^ | 197 | 190 | 190 | 190 | 193 | 193 | 186 | 186 | 199 | 187 | 223 | 223 | 201 | 198 | 169 | 157 | 128 | 125 | 227 | 221 | 249 | 216 | 233 | 221 | 243 | 207 | 0 | 0 | 1 |
| Voronezh13 | 3.9•10^-14^ | 190 | 190 | 193 | 193 | 193 | 193 | 182 | 178 | 199 | 199 | 232 | 217 | 198 | 189 | 169 | 157 | 125 | 125 | 218 | 218 | 219 | 195 | 233 | 233 | 210 | 180 | 133 | 127 | 1 |
| Voronezh14 | 1.6•10^-17^ | 200 | 200 | 193 | 193 | 202 | 196 | 182 | 178 | 199 | 199 | 232 | 232 | 201 | 198 | 181 | 157 | 131 | 125 | 230 | 224 | 149 | 149 | 233 | 233 | 243 | 243 | 0 | 0 | 1 |
| Voronezh15 | 5.7•10^-16^ | 185 | 185 | 193 | 193 | 202 | 193 | 178 | 178 | 199 | 187 | 232 | 232 | 198 | 192 | 169 | 157 | 125 | 125 | 230 | 224 | 219 | 219 | 233 | 221 | 210 | 210 | 133 | 0 | 1 |
| Voronezh16 | 2.3•10^-17^ | 200 | 200 | 193 | 190 | 199 | 193 | 186 | 178 | 193 | 193 | 232 | 232 | 198 | 189 | 187 | 187 | 131 | 125 | 221 | 218 | 246 | 222 | 227 | 227 | 240 | 213 | 133 | 133 | 1 |
| Voronezh17 | 4.3•10^-13^ | 190 | 190 | 190 | 190 | 193 | 193 | 186 | 186 | 199 | 193 | 232 | 220 | 198 | 198 | 172 | 169 | 131 | 125 | 224 | 221 | 249 | 219 | 233 | 227 | 243 | 210 | 133 | 133 | 1 |
| Voronezh19 | 3.1•10^-16^ | 200 | 190 | 190 | 190 | 190 | 190 | 186 | 178 | 193 | 187 | 247 | 211 | 201 | 198 | 169 | 157 | 131 | 125 | 233 | 224 | 231 | 192 | 227 | 221 | 219 | 180 | 133 | 133 | 1 |
| Voronezh22 | 4.5•10^-16^ | 200 | 188 | 190 | 187 | 190 | 190 | 186 | 186 | 199 | 199 | 235 | 211 | 198 | 192 | 169 | 169 | 125 | 125 | 227 | 224 | 252 | 216 | 233 | 233 | 249 | 210 | 133 | 133 | 2 |
| Voronezh23 | 1.4•10^-18^ | 200 | 200 | 190 | 190 | 193 | 193 | 186 | 182 | 205 | 199 | 235 | 220 | 201 | 198 | 157 | 157 | 131 | 125 | 233 | 218 | 243 | 240 | 239 | 233 | 231 | 228 | 133 | 133 | 1 |
| Voronezh25 | 4.0•10^-24^ | 200 | 190 | 187 | 187 | 199 | 193 | 186 | 186 | 187 | 187 | 235 | 223 | 204 | 195 | 174 | 171 | 131 | 125 | 230 | 218 | 210 | 177 | 221 | 221 | 174 | 165 | 127 | 127 | 2 |
| Voronezh27 | 7.3•10^-18^ | 202 | 193 | 190 | 187 | 190 | 190 | 186 | 182 | 199 | 199 | 235 | 211 | 201 | 189 | 169 | 163 | 125 | 125 | 233 | 218 | 216 | 210 | 233 | 233 | 210 | 195 | 0 | 0 | 2 |
| Voronezh28 | 3.0•10^-18^ | 190 | 188 | 187 | 187 | 190 | 190 | 186 | 178 | 199 | 199 | 235 | 235 | 201 | 189 | 169 | 157 | 143 | 131 | 233 | 233 | 237 | 222 | 233 | 233 | 225 | 213 | 133 | 0 | 3 |
| Voronezh29 | 4.0•10^-14^ | 200 | 188 | 190 | 190 | 199 | 196 | 186 | 178 | 199 | 199 | 232 | 211 | 198 | 189 | 186 | 169 | 131 | 125 | 233 | 224 | 219 | 210 | 233 | 233 | 210 | 195 | 133 | 133 | 1 |
| Voronezh30 | 5.0•10^-21^ | 200 | 200 | 187 | 187 | 193 | 193 | 185 | 178 | 187 | 187 | 223 | 223 | 198 | 189 | 174 | 174 | 128 | 125 | 227 | 224 | 219 | 201 | 221 | 221 | 210 | 186 | 0 | 0 | 1 |
| Yoshkar-Ola6 | 6.4•10^-15^ | 190 | 185 | 193 | 193 | 199 | 190 | 182 | 178 | 199 | 199 | 226 | 220 | 201 | 198 | 187 | 157 | 131 | 125 | 233 | 227 | 219 | 192 | 233 | 233 | 207 | 180 | 127 | 0 | 1 |
| Yoshkar-Ola11 | 7.1•10^-11^ | 190 | 185 | 193 | 193 | 190 | 190 | 186 | 186 | 199 | 199 | 235 | 235 | 198 | 198 | 169 | 157 | 131 | 125 | 233 | 233 | 210 | 210 | 233 | 233 | 198 | 198 | 133 | 0 | 1 |
| Yoshkar-Ola14 | 7.3•10^-10^ | 185 | 185 | 190 | 190 | 190 | 190 | 186 | 186 | 199 | 199 | 241 | 235 | 198 | 198 | 190 | 163 | 131 | 131 | 227 | 224 | 249 | 210 | 233 | 233 | 243 | 198 | 133 | 133 | 1 |
| Yoshkar-Ola15 | 1.0•10^-9^ | 200 | 200 | 190 | 190 | 190 | 190 | 186 | 186 | 199 | 187 | 235 | 235 | 201 | 198 | 181 | 163 | 131 | 125 | 224 | 224 | 222 | 219 | 233 | 221 | 210 | 207 | 133 | 133 | 1 |
| Yoshkar-Ola16 | 1.3•10^-14^ | 190 | 185 | 193 | 193 | 199 | 190 | 182 | 178 | 199 | 199 | 226 | 220 | 201 | 198 | 184 | 157 | 131 | 125 | 233 | 227 | 219 | 195 | 233 | 233 | 207 | 180 | 127 | 0 | 1 |
| Yoshkar-Ola18 | 7.3•10^-10^ | 185 | 185 | 190 | 190 | 190 | 190 | 186 | 186 | 199 | 199 | 241 | 235 | 198 | 198 | 190 | 163 | 131 | 125 | 227 | 224 | 249 | 210 | 233 | 233 | 243 | 198 | 133 | 133 | 9 |
| Yoshkar-Ola21 | 2.5•10^-11^ | 190 | 185 | 193 | 190 | 190 | 190 | 186 | 182 | 199 | 199 | 235 | 235 | 201 | 198 | 184 | 169 | 128 | 128 | 227 | 224 | 210 | 210 | 233 | 233 | 198 | 198 | 133 | 133 | 1 |
| Yoshkar-Ola22 | 8.1•10^-12^ | 200 | 190 | 190 | 190 | 190 | 190 | 186 | 186 | 199 | 193 | 235 | 211 | 204 | 198 | 169 | 163 | 131 | 131 | 227 | 227 | 249 | 219 | 233 | 227 | 243 | 207 | 133 | 133 | 1 |
| Yoshkar-Ola24 | 1.0•10^-9^ | 200 | 200 | 190 | 190 | 190 | 190 | 186 | 186 | 199 | 187 | 235 | 235 | 201 | 198 | 181 | 163 | 131 | 131 | 224 | 224 | 222 | 219 | 233 | 221 | 210 | 207 | 133 | 133 | 5 |
| Yoshkar-Ola25 | 2.8•10^-12^ | 200 | 190 | 190 | 190 | 193 | 193 | 186 | 178 | 199 | 193 | 235 | 217 | 201 | 198 | 169 | 157 | 125 | 125 | 224 | 224 | 222 | 219 | 233 | 221 | 210 | 207 | 133 | 133 | 1 |
| Yoshkar-Ola28 | 5.2•10^-14^ | 200 | 197 | 190 | 190 | 193 | 193 | 178 | 178 | 199 | 199 | 235 | 214 | 201 | 189 | 169 | 157 | 131 | 131 | 227 | 227 | 228 | 219 | 233 | 233 | 216 | 207 | 133 | 133 | 1 |
| Yoshkar-Ola30 | 5.3•10^-9^ | 200 | 200 | 190 | 190 | 190 | 190 | 186 | 186 | 199 | 187 | 235 | 235 | 201 | 198 | 181 | 163 | 131 | 131 | 227 | 227 | 222 | 219 | 233 | 233 | 210 | 207 | 133 | 133 | 2 |
| Yoshkar-Ola32 | 1.3•10^-17^ | 190 | 187 | 190 | 190 | 199 | 193 | 186 | 186 | 199 | 175 | 235 | 232 | 201 | 189 | 169 | 157 | 131 | 125 | 218 | 218 | 225 | 219 | 233 | 209 | 213 | 207 | 127 | 0 | 7 |

Note.

^1^ GP – probability of appearance of genotype in a sample.

^2^ NR – number of ramets found.

Diploid genotypes are coded by pairs of numbers corresponding to molecular weight of alleles at microsatellite loci. “0” is a code for null alleles (variant non-amplified by PCR).
